# Importin subunit beta‐1 mediates ERK5 nuclear translocation, and its inhibition synergizes with ERK5 kinase inhibitors in reducing cancer cell proliferation

**DOI:** 10.1002/1878-0261.13674

**Published:** 2024-07-04

**Authors:** Zoe Lombardi, Lucia Gardini, Anatolii V. Kashchuk, Alessio Menconi, Matteo Lulli, Ignazia Tusa, Alessandro Tubita, Luisa Maresca, Barbara Stecca, Marco Capitanio, Elisabetta Rovida

**Affiliations:** ^1^ Department of Clinical and Experimental Biomedical Sciences University of Florence Italy; ^2^ National Institute of Optics, National Research Council Florence Italy; ^3^ European Laboratory of Non‐Linear Spectroscopy (LENS) Florence Italy; ^4^ Department of Physics and Astronomy University of Florence Italy; ^5^ Core Research Laboratory – Institute for Cancer Research and Prevention (ISPRO) Florence Italy

**Keywords:** combined therapy, ivermectin, *KPNB1*, *MAPK7*, nuclear import

## Abstract

The mitogen‐activated protein kinase (MAPK) extracellular signal‐regulated kinase 5 (ERK5) is emerging as a promising target in cancer. Indeed, alterations of the MEK5/ERK5 pathway are present in many types of cancer, including melanoma. One of the key events in MAPK signalling is MAPK nuclear translocation and its subsequent regulation of gene expression. Likewise, the effects of ERK5 in supporting cancer cell proliferation have been linked to its nuclear localization. Despite many processes regulating ERK5 nuclear translocation having been determined, the nuclear transporters involved have not yet been identified. Here, we investigated the role of importin subunit alpha (α importin) and importin subunit beta‐1 (importin β1) in ERK5 nuclear shuttling to identify additional targets for cancer treatment. Either importin β1 knockdown or the α/β1 importin inhibitor ivermectin reduced the nuclear amount of overexpressed and endogenous ERK5 in HEK293T and A375 melanoma cells, respectively. These results were confirmed in single‐molecule microscopy in HeLa cells. Moreover, immunofluorescence analysis showed that ivermectin impairs epidermal growth factor (EGF)‐induced ERK5 nuclear shuttling in HeLa cells. Both co‐immunoprecipitation experiments and proximity ligation assay provided evidence that ERK5 and importin β1 interact and that this interaction is further induced by EGF administration and prevented by ivermectin treatment. The combination of ivermectin and the ERK5 inhibitor AX15836 synergistically reduced cell viability and colony formation ability in A375 and HeLa cells and was more effective than single treatments in preventing the growth of A375 and HeLa spheroids. The increased reduction of cell viability upon the same combination was also observed in patient‐derived metastatic melanoma cells. The combination of ivermectin and ERK5 inhibitors other than AX15836 provided similar effects on cell viability. The identification of importin β1 as the nuclear transporter of ERK5 may be exploited for additional ERK5‐inhibiting strategies for cancer therapy.

## Graphical abstract

We identified importin subunit beta‐1 (importin β1) as the nuclear transporter involved in ERK5 nuclear translocation. Combinations of the α/β1 importin inhibitor ivermectin and ERK5 inhibitors were more effective than single treatments in reducing the growth of cancer models. Identification of importin β1 as the nuclear transporter of ERK5 may be exploited for additional ERK5‐inhibiting strategies for cancer therapy.

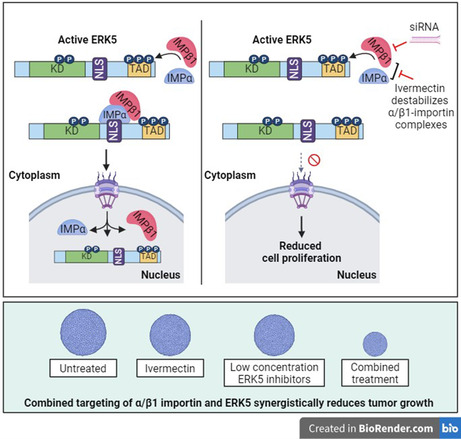

**Abbreviations**
ATCC: American Type Culture Collection
BMK1: big mitogen‐activated protein kinase
DMEM: Dulbecco's Modified Eagle's Medium
DMSO: dimethyl sulphoxide
EGF: epidermal growth factor
ERK5: extracellular signal‐regulated kinase 5
FBS: foetal bovine serum
IP: immunoprecipitation
IVM: ivermectin
KD: knockdown
*KPNB1*
: karyopherin subunit beta‐1
MAPK: mitogen‐activated protein kinase
NLS: nuclear localization signal
NPC: nuclear pore complex
PI: propidium iodide
PLA: proximity ligation assay
SDS/PAGE: sodium dodecyl sulphate/polyacrylamide gel electrophoresis
SD: standard deviation

## 1 Introduction

The extracellular signal‐regulated kinase 5 (ERK5), also named big mitogen‐activated protein kinase1 (BMK1) because it is two times longer than the other mitogen‐activated protein kinases (MAPKs) [1, 2], possesses an N‐terminal kinase domain highly homologous to that of ERK1 and ERK2, and a long C‐terminal part, which includes a transcriptional transactivation domain and a bipartite classical nuclear localization signal (cNLS) [3]. In the absence of stimulation, the N‐terminus of ERK5 is bound to the C‐terminus, thus supporting cytoplasmic ERK5 retention, while the activating phosphorylation of the N‐terminal half by MEK5 determines the disruption of the binding, allowing for nuclear import of ERK5 [4]. Once in the nucleus, ERK5 exerts its transactivation activity on a number of transcription factors, including MEF2 transcription factors through a MEF2‐interacting domain [5]. ERK5 activation is achieved through MEK5‐dependent phosphorylation of Thr219/Tyr221 in the kinase domain that leads to the exposition of the cNLS and subsequent ERK5 nuclear translocation and transcriptional activity that supports cell proliferation [4, 6, 7]. Interestingly, endogenous ERK5 may localize in the nucleus of resting HeLa and melanoma cells [8, 9].

Increased expression and activation of MEK5/ERK5 have been reported in several cancers, in which they are associated with advanced stages and metastases [10]. Therefore, MEK5/ERK5 targeting is increasingly taken into consideration for cancer treatment, and a number of MEK5 or ERK5 inhibitors have been developed [11, 12, 13]. However, it has been reported that some ERK5 kinase inhibitors may induce a paradoxical activation of ERK5 by increasing its nuclear shuttling and transcriptional transactivation activity. Targeting ERK5 nuclear translocation in cancer cells could thus be a valid approach *per se* or in combination with ERK5 kinase inhibitors.

Small proteins can enter the nucleus by simple diffusion through the nuclear pores, whereas proteins with a higher molecular weight (more than about 60 kDa), including some MAPKs, are actively transported by nuclear transporters. Many of these belong to the karyopherin β (or importin β) and karyopherin α (importin α) superfamily. Additionally, it is well known that cNLS on cargo proteins are recognized by importin α, which, in turn, heterodimerizes with the importin β1 subunit. Importin β1, in turn, is able to interact with components of the nuclear pore complex (NPC), called nucleoporins, so that the resulting cargo‐α/β1‐importin‐trimer is imported into the nucleus [14]. Based on all above and on the presence of a cNLS in ERK5 sequence [5, 15], it has been suggested that ERK5 shuttles into the nucleus via α/β importin [16]. This work was undertaken to investigate on this hypothesis, with the aim of identifying the nuclear transporters involved in ERK5 nuclear shuttling, in view of their targeting to prevent this crucial event for ERK5 activities.

## 2 Materials and methods

### 2.1 Cell cultures

A375 melanoma cells (CRL‐1619), HEK‐293T (CRL‐3216) and HeLa cells (CRM‐CCL‐2) were obtained from ATCC (Manassas, VA, USA) in 2014. HUH‐7 hepatocellular carcinoma cells (CVCL_0336) were kindly gifted by Prof Fabio Marra (AOUC, Firenze, Italy) in 2012. SK‐Mel‐5 melanoma cells were kindly provided by Dr Laura Poliseno (CRL‐ISPRO, Pisa, Italy). Cells were maintained in Dulbecco's Modified Eagle's Medium (DMEM) with 10% heat‐inactivated foetal bovine serum (FBS; complete medium), 2 mm glutamine, 50 U·mL^−1^ penicillin and 50 mg·mL^−1^ streptomycin (Euroclone, Paignton, UK). Cell lines were yearly authenticated by cell profiling (Promega PowerPlex Fusion System kit; BMR Genomics s.r.l; Padova, Italy). The presence of mycoplasma was periodically tested by PCR. Short‐term melanoma cultures (Me53, Me58 and Me59) were established as previously described [17] from metastatic cutaneous melanomas obtained from Plastic and Reconstructive Surgery Unit of the S.M. Annunziata Hospital (Florence, Italy). The study and protocols were approved by the local Independent Ethical Committee (Comitato Etico Regionale per la Sperimentazione Clinica della Regione Toscana, Area Vasta Centro; Protocol 16922_bio). The experiments were undertaken with the understanding and written consent of each subject. The study methodologies conformed to the standards set by the Declaration of Helsinki. Samples were collected from February 2021 to March 2023.

### 2.2 Drugs

Ivermectin (α/β1 importin inhibitor, IVM) [18] and the ERK5 inhibitors XMD8‐92 [11], JWG‐071 [12] and AX15836 [13] were from MedChemExpress LLC (Monmouth Junction, NJ, USA).

### 2.3 Cell lysis and western blot

Total cell lysates using Laemmli buffer and nucleoplasm fractions were obtained as previously described [9]. Proteins were separated as previously reported [19]. Chromatin‐bound fractions were obtained with a subcellular protein fractionation kit (Thermo Fisher Scientific, Waltham, MA, USA). Images were quantified with imagej software. Antibodies are listed in Table S1.

### 2.4 Co‐immunoprecipitation (Co‐IP)

HeLa cells transfected with ERK5/MEK5DD and A375 melanoma cells were lysed in Co‐IP buffer (50 mm Tris‐HCl, pH 7.4, NaCl 150 mm, 1 mm EDTA, Triton X‐100 1%, with 1 mm Na_3_VO_4_, 20 mm Na_4_P_2_O_7_, 1 mm PMSF, 0.1 U·mL^−1^ Aprotinin, 1 mm TPKC). Samples were then incubated with primary antibodies (listed in Table S1) for 2 h at 4 °C and then with protein A/G‐Sepharose beads (Santa Cruz, Dallas, TX, USA) overnight at 4 °C. After several washes with Co‐IP buffer, bound proteins were eluted by adding Laemmli buffer 4× and incubating at 99 °C and analysed by SDS/PAGE. Western blot was then performed either reblotting the same membrane with different antibodies or running the same samples in parallel gels. IgG content was used as a loading control.

### 2.5 Immunofluorescence and proximity ligation assay (PLA)

HeLa cells were plated on glass coverslips and incubated for 24 h in DMEM/10%FBS, and then, medium was replaced with DMEM/0%FBS alone or with IVM 5 μm. After 24 h, cells were treated with EGF (ImmunoTools, Friesoythe, Germany) 100 ng·mL^−1^ for 30 min and fixed with 4% paraformaldehyde (10 min, room temperature). Cells were permeabilized (0.2% Triton X‐100) and incubated with 10% horse serum in PBS/1% BSA for 45 min. Incubation with primary antibody (overnight, 4 °C) and with Cy2‐ or Cy3‐labeled secondary antibodies was performed. Cell nuclei were labelled with DAPI (Invitrogen, Waltham, MA, USA). Images were taken with a Leica TCS SP8 scanning confocal microscopy system with a Plan Apo 60× objective (Leica Microsystems, Mannheim, Germany).

For PLA, HeLa cells were seeded in 8‐well chambered coverslips (Ibidi, Gräfelfing, Germany) in DMEM/10%FBS and incubated for 24 h, and then, medium was replaced with DMEM/0%FBS. After 24 h of starvation, cells were treated with EGF 100 ng·mL^−1^ for 30 min and fixed with 4% paraformaldehyde (10 min, room temperature). Cells were permeabilized (0.2% Triton X‐100) and incubated with Blocking buffer included in PLA kit Naveni™TriFlex Cell MR (Navinci, Husargatan, Sweden). Incubation with primary antibody (overnight, 4 °C) and PLA assay were performed following manufacturer's procedure. Cell nuclei were labelled with DAPI (Invitrogen). Images were taken with a Leica TCS SP8 scanning confocal microscopy system with a Plan Apo 60× objective (Leica Microsystems). Quantification of the PLA signal was obtained by analysing the integrated optical density from five images/condition.

### 2.6 Plasmids and transfection

pcDNA3.1‐HA‐ERK5wt construct was a kind gift from Atanasio Pandiella (CIC, Salamanca, Spain). The pcMV5‐MEK5DD‐HA (a constitutively active form of MEK5) was generously provided by Jiing‐Dwan Lee (Scripps Institute, La Jolla, CA, USA). The ERK5‐HaloTag‐HA‐expressing vector has been developed in our lab. To this end, ERK5 full‐length sequence was amplified from the pcDNA HA‐ERK5 plasmid using the primers below: the forward (FW) primer allowed the insertion of the Kozac consensus sequence; the reverse (RV) primer allowed the amplification of the complete ERK5 sequence excluding the stop codon. The amplified sequence was cloned into the pcDNA3.1(+) plasmid using the BamHI/NotI restriction sites. The obtained plasmid is termed pCDNA3.1 ERK5.

- ERK5 Kozac FW: CGGGATCCCCACCATGGCCGAGCCTCTGA
- ERK5 w/o stop RV: ATAAGAATGCGGCCGCGGGGTCCTGGAGGTCAGGC

HaloTag sequence, having at 3′ the coding sequence for the 3xHA‐Tag and the stop codon, was amplified from the plasmid pFC20A using the primers below. The amplified sequence was cloned into the pCDNA3.1 ERK5 vector by forced cloning using the XhoI/XbaI restriction sites. The obtained plasmid is termed pCDNA3.1 ERK5 Halo 3xHA.

- Halo w/o Kozac FW: CCGCTCGAGGGGAAATCGGTACTGGCTTTCCA
- Halo w stop 3xHA RV: GCTCTAGATTAAGCGTAGTCAGGTACGTCGTAAGGGTATTCCGCAGCGTAATCCGGAACGTCGTACGGATATTCCGCCGCGTAGTCTGGAACGTCATATGGGTAACCGGAAATCTCCAGAGT

Agarose gel electrophoresis after the restriction enzyme digestion of the obtained plasmid was performed to verify cloning accuracy.

For transient transfection, cells were plated on six‐well dishes (3 × 10^5^ cells per well) and transfected after 24 h with a total amount of 2 μg of plasmid DNA using JetPEI reagent (Polypus Transfection from Euroclone, Paignton, UK), following manufacturer's instructions. Cells were lysed after 24–48 h. IVM was added 18 h before lysis.

### 2.7 Cell viability and colony formation assay

Cell viability and apoptosis were measured by MTT and Annexin V/propidium iodide staining assays, respectively, as reported previously [19]. To determine primary melanoma cell viability, Me53, Me58 and Me59 cells were seeded in 96‐well plates and treated with vehicle (dimethyl sulphoxide, DMSO), IVM or AX15836 or the combination. After 72 h, cells were fixed with 4% PFA and stained with crystal violet. The cells were then de‐stained with 10% acetic acid, and absorbance was read at 590 nm on Victor X5 (PerkinElmer, Waltham, MA, USA). Colony assay was performed as previously described [19]. Colonies (i.e. more than 50 cells [20]) were counted following crystal violet staining after 7 days.

### 2.8 3D spheroid cultures and live dead cell assay

Cells were seeded in 96‐well plate that had been previously coated with a base coating a solution of 1.5% agarose in water, in a number of 1000 cells per well in 0.22 μm‐filtered DMEM/10%FBS. After 72 h (time 0), spheroids were treated with IVM and/or AX15836 and incubated for additional 5 days. Photos were taken at the indicated times, and spheroid volume was then quantified with imagej [Volume = 0.5*L*W ^2^, *L* = length (major axis) *W* = width (minor axis)]. For live dead assay, 4 days post‐time 0 media was removed, and spheroids were washed once with PBS and stained by adding DMEM without phenol red containing 4 μm Calcein AM (Santa Cruz Biotechnology, Inc., Dallas, TX, USA) and 2 μm propidium iodide (PI) (Sigma‐Merck, St. Louis, MO, USA) for 30 min at 37 °C. Images were taken after transferring spheroids on glass coverslips with a Leica TCS SP8 scanning confocal microscopy system with a Plan Apo 60× objective (Leica Microsystems). The percentage of red‐positive (PI, dead) or green‐positive (Calcein AM, alive) cells in the spheroid structure has been quantified with Analyze Particles Plugin available on imagej software.

### 2.9 Importin β1 knockdown

Cells were transfected with 50 nm KPNB1‐targeting siRNA or siCONTROL non‐targeting siRNA (siNT) as previously reported [19]. Cells were seeded in complete medium without antibiotics and incubated for 24 h until they reached 50% confluence. Transfection was then performed with Lipofectamine 2000 (Thermo Fisher Scientific) and siRNA for KPNB1 or siNT following the manufacturer's instructions. Used siRNAs (Sigma‐Merck) were siKPNB1‐1‐SASI_HS01_0010‐1673 and siKPNB1‐2‐SASI_HS02_0033‐3523 together with a siNT (SIC001).

### 2.10 Super‐resolution imaging

Single molecule localization analysis was performed as previously described [21, 22]. In particular, HeLa cells were seeded (18 000 cell per well) on cover glass (18 × 18 mm) coated with polylysine for 1 h at 37 °C and then transfected with ERK5‐HaloTag‐HA‐expressing vector using JetPEI reagent (Polypus Transfection, Euroclone, Milan, Italy) in DMEM/10%FBS without antibiotics following manufacturer's procedure. After 24 h, cells were washed once with Leibovitz medium and then labelled with JaneliaFluor646 (diluted in Leibovitz medium to 0.05 μm final concentration) for 15 min at 37 °C. Cells were then washed once and cover glass was placed in a microscope chamber and covered with Leibovitz medium for living imaging. Single molecule localization imaging of ERK5‐HaloTag‐JaneliaFluor646 was performed on an inverted wide‐field fluorescence microscope (Nikon ECLIPSE TE300) with 643 nm excitation laser and 405 nm activation laser. Excitation and activation were performed with inclined illumination through a TIRF 60× objective (Nikon 60×, oil immersion, NA 1.49 TIRF) to optimize the signal‐to‐background ratio. Emitted fluorescence was collected through the same objective and imaged on an EMCCD Camera (Andor iXon X3) after an additional 3× magnification. Full field of view is 40 × 40 μm^2^ with 80 nm pixel size. A total of 3000 frames were acquired for each cell, with 30 ms exposure time and 400 EMgain. Activation of fluorophores was performed at frames 100, 1000 and 2000 with 200 ms exposure to 405 nm laser light (10 μWon the sample). A brightfield image of each cell was recorded for masking of nucleus and cytoplasm. Masks are drawn manually using custom and built‐in tools in matlab. To avoid errors introduced by uneven illumination at the edges of the image, the masks are limited to a centred circle of *R* = 300 pixels. ERK5‐HaloTag molecules were localized using thunderstorm [23] plugin for imagej with the following parameters: Image filtering: Wavelet filter (B‐Spline), order = 8, scale = 2; Approximate localization: Local maximum, PIT = std (Wave.F1), Connectivity = 8; Sub‐pixel localization: Radial symmetry, *R* = 3 px. A 200 of post‐activation and 20 pre‐activation frames are omitted which gives a total of 2280 images included in the analysis for each measurement. The ratio *r* of the densities of detected ERK5 molecules (nucleus to cytoplasm) is determined as:

$$r=\frac{N_{n}/S_{n}}{N_{c}/S_{c}}$$

where *N* is the number of detected molecules in a masked region, *S* is the area of the masked region, and indices *n* and *c* indicate the nucleus and the cytoplasm, respectively.

### 2.11 Statistical analysis

Data represent mean or ±SD values calculated on at least three independent experiments. *P* values were calculated using Student's *t*‐test or one‐way analysis of variance (ANOVA, multiple comparison using Bonferroni's correction). A two‐tailed value of *P* < 0.05 was considered statistically significant.

## 3 Results

### 3.1 α/β1 importin mediates ERK5 nuclear shuttling

**Fig. 1:**
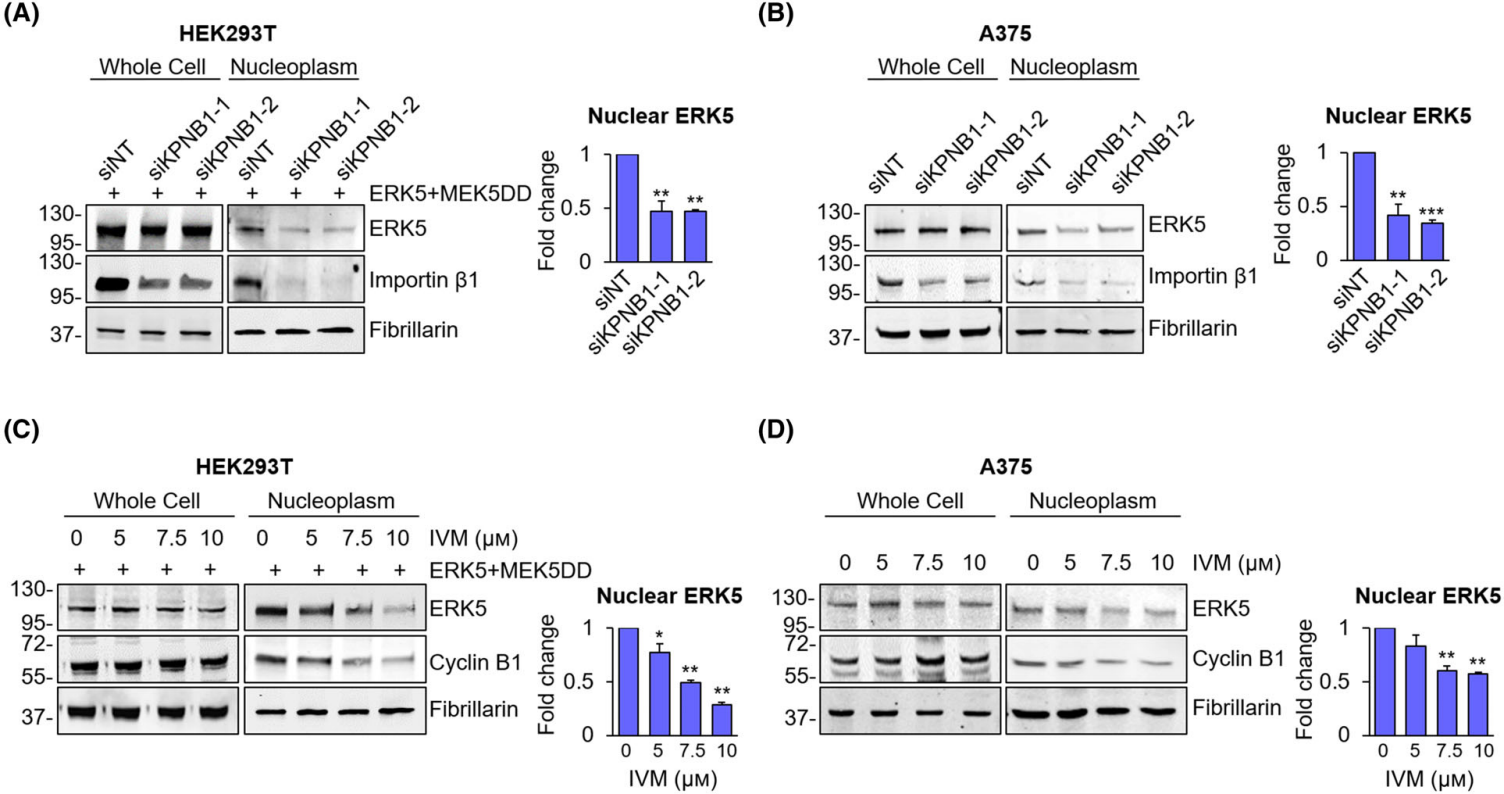
KPNB1 inhibition reduces the amount of nuclear ERK5. (A, B) HEK293T overexpressing ERK5 and the constitutively active form of MEK5 (ERK5 + MEK5DD) (A) or A375 (B) cells transfected with two siRNA targeting KPNB1 (siKPNB1‐1 or siKPNB1‐2) or with non‐targeting control siRNA (siNT) were lysed, and western blot was performed on whole cell lysates or nuclear extracts with the indicated antibodies. Migration of molecular weight markers is indicated on the left (kDa). The graphs show average densitometric values ± SD of nuclear ERK5 protein levels normalized for fibrillarin content (*n* = 3). *P* values calculated using Student's *t*‐test refer to differences with respect to siNT‐treated control cells. **, *P* < 0.01 and ***, *P* < 0.001. (C, D) HEK293T overexpressing ERK5 and the constitutively active form of MEK5 (ERK5 + MEK5DD) (C) or A375 (D) cells treated for 24 h with DMSO, used as vehicle, or increasing concentration of ivermectin (IVM) were lysed, and western blot was performed on whole cell lysates or nuclear extracts with the indicated antibodies. The graphs show average densitometric values ± SD of nuclear ERK5 protein levels normalized for fibrillarin content (*n* = 3). *P* values calculated using Student's *t*‐test refer to differences with respect to vehicle‐treated control cells. *, *P* < 0.05 and **, *P* < 0.01.

The presence of a cNLS in ERK5 sequence [4] prompted us to investigate on a possible involvement of α/β1 importin in ERK5 nuclear translocation. To this end, we knocked down importin β1, encoded by the *KPNB1* gene, using two different siRNAs similarly effective in reducing importin β1 protein levels (Fig. S1A,B). Western blot analysis showed that the amount of ERK5 in the nucleus, boosted by overexpression of ERK5 and of the constitutively active form of its activator MEK5 (MEK5DD) in HEK293T cells, was reduced by 50% following KPNB1 knockdown (KD) with respect to non‐targeting siRNA‐treated cells (siNT) (Fig. 1A). This effect was not due to a decrease of ERK5 protein in the cells, as witnessed by the lack of changes in whole cell lysates, and was also observed in the absence of MEK5DD‐induced activation of ERK5 (Fig. S1C). Additionally, KPNB1 KD was able to halve the amount of endogenous ERK5 in the nucleus in A375 melanoma cells with respect to siNT‐treated ones (Fig. 1B). A375 cells have been chosen as a model of melanoma, in which we previously demonstrated that oncogenic BRAF supports ERK5 nuclear localization in routinely cultured cells, thus supporting cell proliferation [9].

In order to identify pharmacological treatments able to prevent ERK5 nuclear localization, we used the α/β1 inhibitor IVM. IVM reduced the amount of nuclear ERK5 in a dose‐dependent manner in both HEK293T overexpressing ERK5/MEK5DD and A375 (endogenous ERK5) cells (Fig. 1C,D). Confirmation of the inhibitory activity of IVM on α/β1 importin is witnessed by the reduction of nuclear cyclin B1 protein, a known target of α/β1 importin [24]. Of note, IVM did not change the amount of ERK5 nor of cyclin B1 in whole cell lysates (Fig. 1C,D). Interestingly, in both HEK293T overexpressing ERK5/MEK5DD and A375 cells, treatment with IVM robustly reduced the amount of ERK5 linked to chromatin ([9] Fig. S2A). Similarly to what observed upon KPNB1 KD (Fig. S1C), IVM reduced the amount of nuclear ERK5 also in the absence of MEK5DD‐induced activation (Fig. S2B). As expected, the activity of ERK5 in the nucleoplasm is higher upon MEK5DD overexpression as witnessed by increased levels of KLF‐2 (whose expression is decreased upon IVM treatment), despite the total amount of ERK5 is decreased, as previously shown [9].

**Fig. 2:**
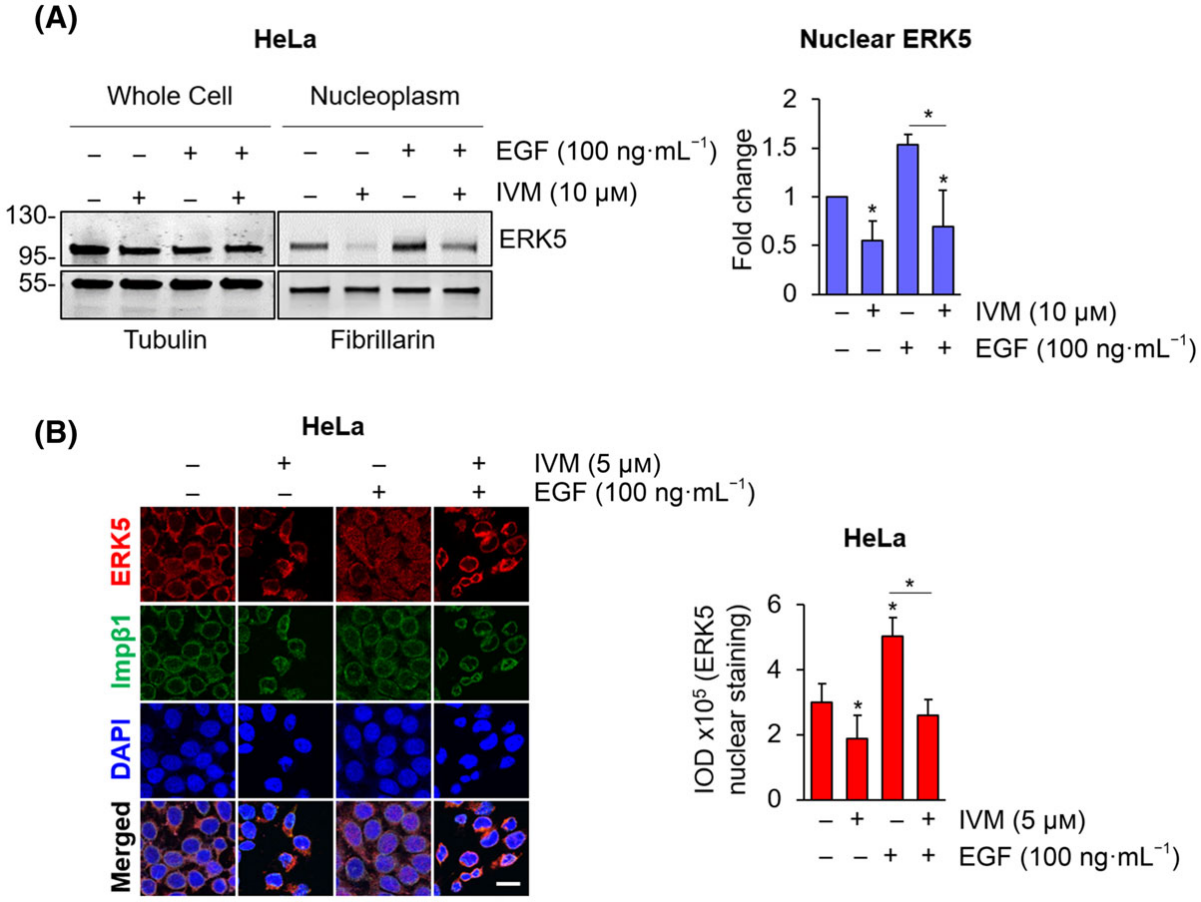
Ivermectin prevents EGF‐induced nuclear translocation of ERK5. (A) 24‐h starved HeLa cells were treated with DMSO, used as vehicle, or 10 μm ivermectin (IVM) for 24 h and then with 100 ng·mL^−1^ epidermal growth factor (EGF) for 30 min or the combination of the two. Cells were then lysed, and western blot was performed on whole cell lysates or nuclear extracts with the indicated antibodies. Migration of molecular weight markers is indicated on the left (kDa). The graph shows average densitometric values ± SD of nuclear ERK5 protein levels normalized for fibrillarin content (*n* = 3). *P* values calculated using Student's *t*‐test refer to differences with respect to vehicle‐treated cells or between the indicated conditions. *, *P* < 0.05. (B) 24‐h serum‐starved HeLa cells were treated with DMSO, used as vehicle, or 5 μm ivermectin (IVM) for 24 h and then with 100 ng·mL^−1^ epidermal growth factor (EGF) for 15 min or left untreated. Immunofluorescence analysis was performed staining ERK5 (red) and importin β1 (Imp β1, green). Confocal images were analysed to quantify ERK5 nuclear staining, represented in the graph as integrated optical density (IOD) ± SD (*n* = 3). *P* values calculated using Student's *t*‐test refer to differences with respect to vehicle‐treated cells or between the indicated conditions. *, *P* < 0.05. Scale bar: 20 μm.

In order to provide evidence of the effect of IVM on endogenous ERK5 in both unstimulated and stimulated conditions, we moved to HeLa cells. These cells are extensively used to study ERK5 biology and signalling [13, 25, 26, 27] and are used as a model of endometrial cancer that has been recently included among the neoplasms in which ERK5 targeting reduces tumour growth [28]. Serum‐starved HeLa cells were stimulated or not with EGF, a well‐known inducer of ERK5 activation and nuclear translocation [25], in the presence or absence of IVM. Western blot analysis showed that the amount of ERK5 in the nucleus boosted by EGF was reduced by 50% following IVM treatment (Fig. 2A). To further confirm the effect of IVM on ERK5 nuclear translocation, HeLa cells were stained for ERK5 and importin β1 in immunofluorescence analysis. Confocal imaging showed that EGF stimulation induces ERK5 nuclear accumulation, as expected, whereas IVM treatment reverted this effect (Fig. 2B).

### 3.2 Single molecule analysis allows to confirm the involvement of α/β1 importin in ERK5 nuclear translocation

**Fig. 3:**
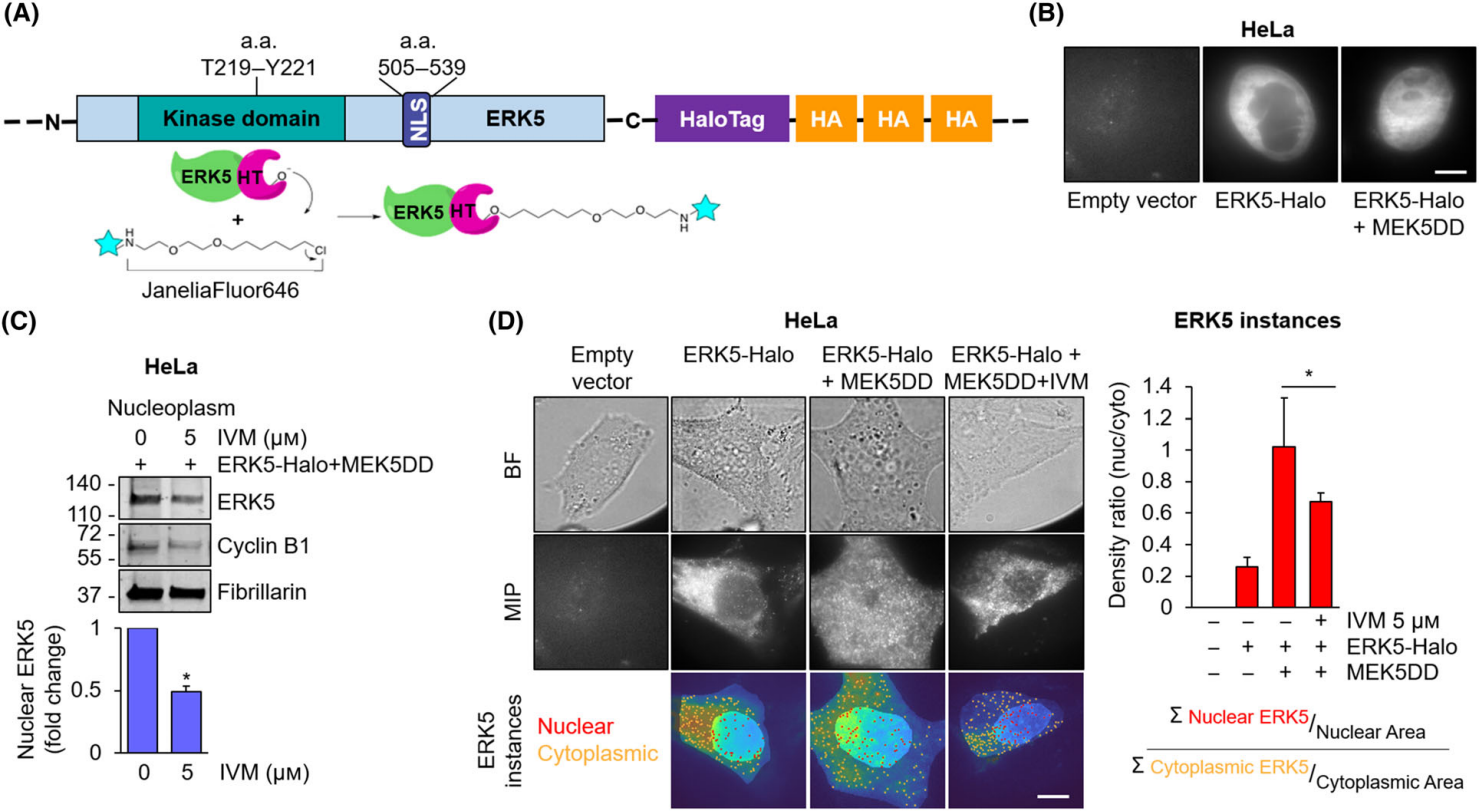
Single molecule analysis confirms the efficacy of ivermectin in reducing ERK5 nuclear translocation. (A) Schematic representation of ERK5‐HaloTag‐JaneliaFluor646 system. Amino acid residues (a.a.); NLS: nuclear localization signal. (B) Confocal imaging of HeLa cells transfected with control empty vector, ERK5‐HaloTag or ERK5‐HaloTag and the constitutively active form of MEK5 (ERK5‐Halo + MEK5DD) and then labelled with JaneliaFluor646. Representative confocal images from three independent experiments are shown. Scale bar: 10 μm. (C) HeLa cells transfected with ERK5‐HaloTag and the constitutively active form of MEK5 (ERK5‐Halo + MEK5DD) were treated with 5 μm ivermectin (IVM) or DMSO, used as vehicle, for 24 h and then lysed. Western blot was then performed on nuclear extracts with the indicated antibodies. Migration of molecular weight markers is indicated on the left (kDa). The graphs show the average densitometric values ± SD of nuclear ERK5 protein levels normalized for fibrillarin content (*n* = 3). *P* values calculated using Student's *t*‐test refer to differences with respect to vehicle‐treated cells. *, *P* < 0.05. (D) Bright‐field images (BF, first row), maximum intensity projection images of super‐resolution videos (MIP, second row) and single frames of the latter overlapped with cytoplasmic and nuclear masks (third row), drawn based on bright‐field images (Fig. S3C), of HeLa cells transfected with an empty vector (first column), ERK5‐HaloTag (second column) or ERK5‐HaloTag and the constitutively active form of MEK5 (ERK5‐Halo + MEK5DD) (third column). Cells were left untreated (1–3 columns) or treated with 5 μm ivermectin (IVM) for 24 h (forth column). All samples were then labelled with JaneliaFluor646. The graph represents nucleus/cytoplasmic density ratios (i.e. the number of ERK5 nuclear particles normalized for the nuclear area/the number of ERK5 cytoplasmic particles normalized for the cytoplasm area) ± SD of ERK5 single molecules detected in the super‐resolution videos in each sample condition. Number of measured cells/samples = 20 (*n* = 3). *P* values calculated using Student's *t*‐test refer to differences between the indicated conditions. *, *P* < 0.05. Scale bar: 10 μm.

To study the effect of IVM on ERK5 nuclear shuttling with another approach, we developed a method to localize single ERK5 molecules using super‐resolution imaging in living cells. To this end, we overexpressed ERK5 linked to the HaloTag (Fig. 3A and Fig. S3A) in HeLa cells (Fig. 3B). The photo‐activatable and cell permeable dye JaneliaFluor646 resulted to selectively bind ERK5‐HaloTag molecules (schematic representation in Fig. 3A), since fluorescent signals were collected in HeLa cells transfected with ERK5‐HaloTag, whereas no signal was collected in empty vector transfected cells (Fig. 3B). Cell fractionation experiments confirmed the efficacy of IVM in reducing the amount of ERK5‐HaloTag in the nucleus of HeLa cells overexpressing ERK5‐HaloTag/MEK5DD with respect to vehicle‐treated cells (Fig. 3C). Treatment with IVM did not affect the amount of ERK5 in whole lysates (Fig. S3B). The effect of IVM on ERK5 nuclear translocation was then investigated using super‐resolution microscopy (Fig. 3D). As expected, quantification of ERK5 instances in the nucleus and in the cytoplasm (Fig. S3C) showed an increase in ERK5 nucleus/cytoplasmic density ratio in HeLa cells overexpressing ERK5‐HaloTag and MEK5DD with respect to those transfected with ERK5‐HaloTag alone (Fig. 3D). In line with the western blot results (Fig. 1C,D), treatment with IVM was able to reduce the nucleus/cytoplasmic density ratio of ERK5 in ERK5‐HaloTag/MEK5DD HeLa cells (Fig. 3D).

### 3.3 ERK5 co‐immunoprecipitates with importin β1

**Fig. 4:**
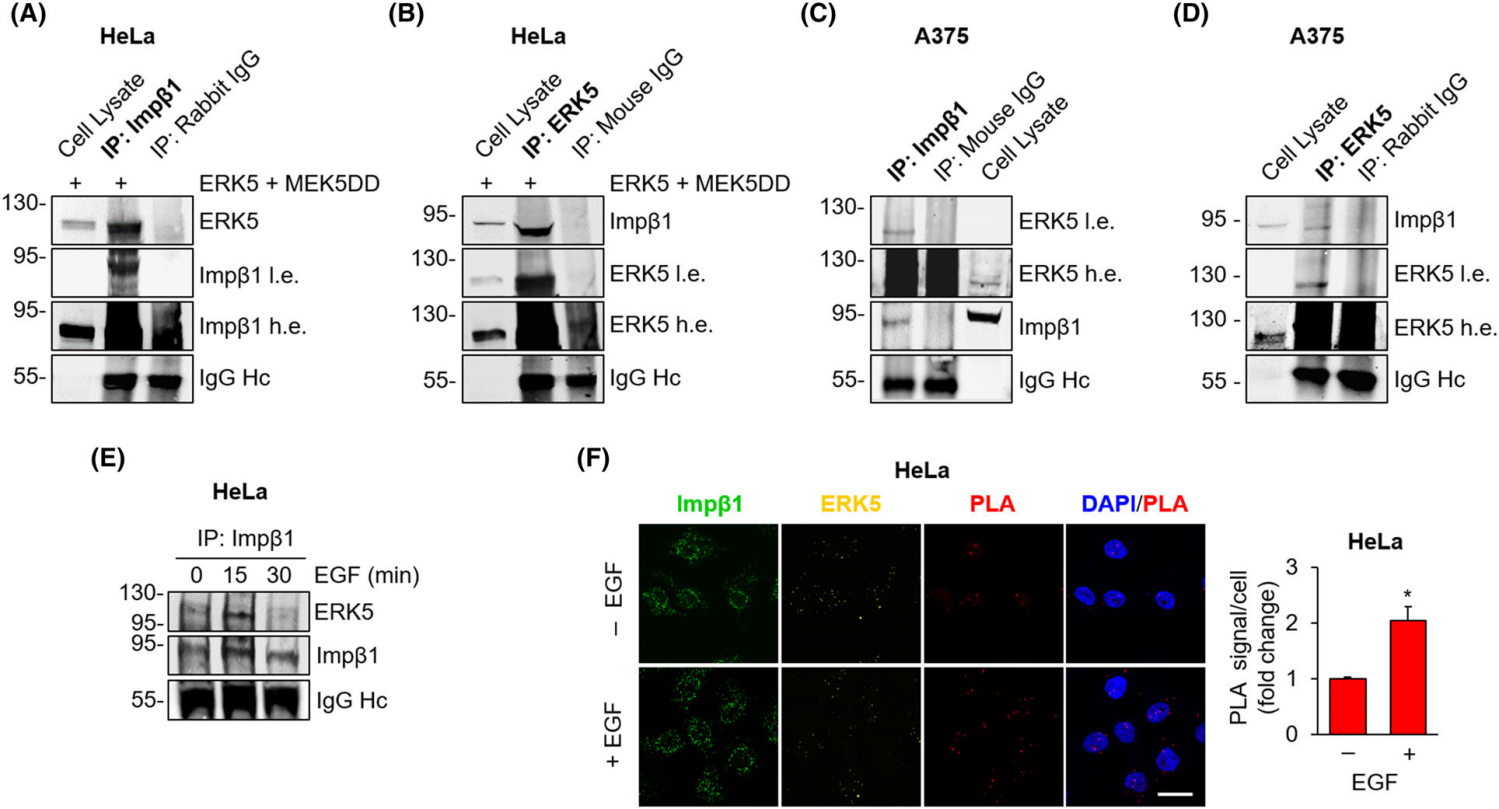
ERK5 interacts with importin β1. (A–D) HeLa cells overexpressing ERK5 and the constitutively active form of MEK5 (ERK5 + MEK5DD) (A, B) or A375 cells (C, D) were lysed, importin β1 (A, C) or ERK5 (B, D) was immunoprecipitated (IP) with specific antibodies as indicated, and western blot was then performed with the indicated antibodies, either reblotting the same membrane with different antibodies or running the same samples in parallel gels. IgG content (Heavy chain, Hc) was used as a loading control. Migration of molecular weight markers is indicated on the left (kDa). Impβ1, importin β1; l.e., low exposure; and h.e. high exposure. Images are representative of three independent experiments. (E) 24‐h serum‐starved HeLa cells were treated with 100 ng·mL^−1^ EGF for the indicated times or left untreated. Cells were then lysed, importin β1 was immunoprecipitated (IP), and western blot was performed with the indicated antibodies. Migration of molecular weight markers is indicated on the left (kDa). Impβ1, importin β1. Images are representative of three independent experiments. (F) 24‐h starved HeLa cells were treated with 100 ng·mL^−1^ EGF for 15 min or left untreated and then stained for ERK5 (yellow) and importin β1 (Impβ1, green). Confocal images were analysed to quantify the proximity ligation assay (PLA) (red) signal. The graph represents average values of PLA signal/cell ± SD. *P* values calculated using Student's *t*‐test refer to differences with respect to untreated cells obtained from three independent experiments. *P* values calculated using Student's *t*‐test refer to differences between the indicated conditions. *, *P* < 0.05. Scale bar: 20 μm.

To prove the interaction between ERK5 and importin β1, we overexpressed ERK5 and MEK5DD to boost ERK5 nuclear shuttling in HeLa cells and performed co‐IP experiments. In western blot, we found ERK5 in the IP for importin β1 (Fig. 4A). The same interaction was revealed upon ERK5 IP and western blot with an anti‐importin β1 antibody (Fig. 4B). The occurrence of an interaction (i.e. co‐IP) between endogenous ERK5 and importin β1 was detected also in A375 melanoma cells (Fig. 3C,D). In order to detect this interaction with and without stimulation, we treated 24‐h starved HeLa cells with EGF. Western blot experiments showed that the amount of endogenous ERK5 immunoprecipitated with importin β1 was increased after 15 min of EGF stimulation with respect to control cells (Fig. 4E). In line with co‐IP results, PLA assay confirmed the existence of an interaction between ERK5 and importin β1 in unstimulated HeLa cells and the increase of this interaction upon EGF stimulation (Fig. 4F).

### 3.4 IVM synergizes with ERK5 inhibitors in reducing cancer cell viability and colony formation ability

**Fig. 5:**
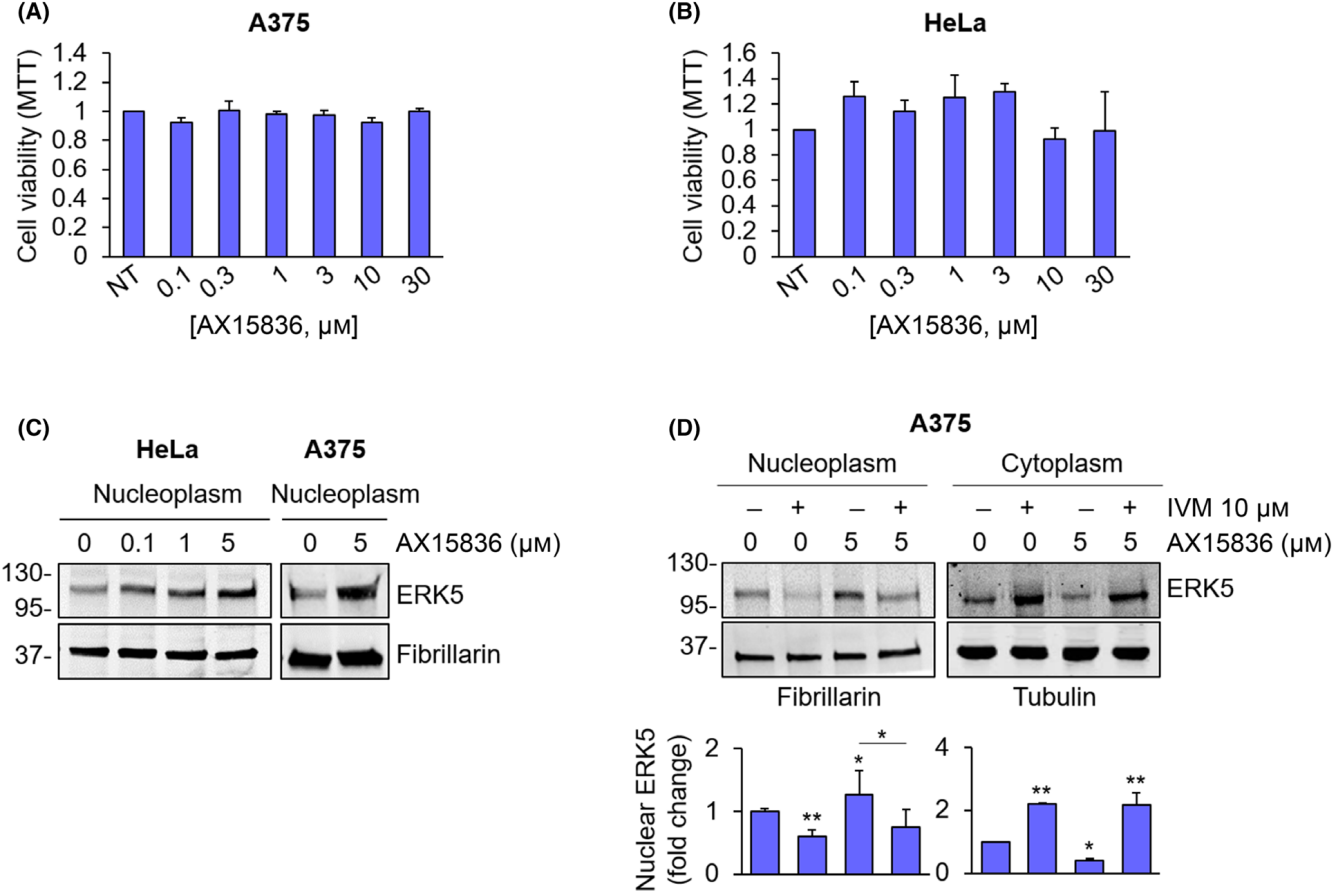
AX15836 does not affect cancer cell proliferation. (A, B) A375 (A) and HeLa cells (B) were treated with increasing concentrations of AX15836, and MTT assays were performed after 72 h. The graphs report average data ± SD (*n* = 3). (C) HeLa and A375 cells treated with DMSO, used as vehicle, or the indicated concentrations of AX15836 for 30 min were lysed, and western blot was performed on nuclear extracts with the indicated antibodies. Migration of molecular weight markers is indicated on the left (kDa). Images are representative of three independent experiments. (D) A375 cells were treated with DMSO, used as vehicle, or ivermectin (IVM) 10 μm for 24 h and then with AX15836 5 μm for 30 min or with the combination of the two drugs were lysed, and western blot was performed on nuclear or cytoplasmic extracts with the indicated antibodies. Migration of molecular weight markers is indicated on the left (kDa). The graphs show average densitometric values ± SD of ERK5 protein levels normalized for fibrillarin or tubulin content (*n* = 3). *P* values calculated with ANOVA refer to differences with respect to vehicle‐treated cells or between indicated conditions. *, *P* < 0.05 and **, *P* < 0.01.

In keeping with data reported in other cell lines [13], MTT experiments showed that the ERK5 inhibitor AX15836 has no effect on the viability of A375 (Fig. 5A) and SK‐Mel‐5 melanoma cells (Fig. S4A), nor on that of HeLa cervical cancer cells (Fig. 5B). The lack of effect on cell proliferation of a number of ERK5 inhibitors, including AX15836, has been ascribed to the paradoxical activation of ERK5 as a consequence of increased nuclear translocation and activation of the transactivation domain in HeLa cells [27]. Here, we confirmed this effect by showing that AX15836 increases the amount of endogenous ERK5 in the nuclear fraction of HeLa cells, in a dose‐dependent manner (Fig. 5C). The same effect was also observed in A375 melanoma cells using 5 μm AX15836 (Fig. 5C). Notably, western blot analysis revealed that IVM prevented the increase of ERK5 in the nucleus after AX15836 treatment in A375 cells (Fig. 5D). The effectiveness of the inhibition of ERK5 phosphorylation upon AX15836 treatment was assessed by gel shift (Fig. 5D).

**Fig. 6:**
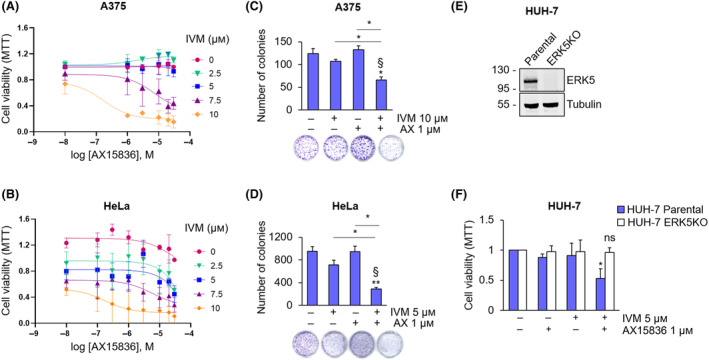
AX15836 inhibits cancer cell proliferation only in combination with ivermectin. (A, B) A375 (A) or HeLa (B) cells were treated with DMSO used as vehicle or increasing concentrations of AX15836 and of ivermectin (IVM) for 72 h, and MTT assay was performed. The graphs report average data ± SD (*n* = 3). (C, D) Colony formation assay was performed with A375 (C) or HeLa (D) cells treated with DMSO used as vehicle (−/−), ivermectin (IVM) or AX15836 (AX) at the indicated concentrations or with their combination for 7 (A375) or 10 (HeLa) days. The graphs report average data ± SD (*n* = 3). *P* values calculated using ANOVA refer to differences with respect to vehicle‐treated cells or between the indicated conditions. *, *P* < 0.05 and **, *P* < 0.01. § indicates synergistic effect (bliss test > 0) with respect to single treatments. (E) Parental and ERK5‐KO HUH‐7 cells were lysed, and western blot was performed with the indicated antibodies. Migration of molecular weight markers is indicated on the left (kDa). (F) Parental and ERK5‐KO HUH‐7 cells were treated with DMSO used as vehicle, AX15836 (AX) or ivermectin (IVM) at the indicated concentration for 72 h, and MTT assay was performed. The graphs report average data ± SD (*n* = 3). *P* values calculated using Student's *t*‐test refer to differences with respect to vehicle‐cells. *, *P* < 0.05; ns, not significant.

Based on the above results, we tested the combination of AX15836 and IVM on cell viability and found that 10 μm IVM alone reduced cell proliferation by 27% (A375) and 44% (HeLa), depending on cell types (Fig. 6A,B), in keeping with its activity in preventing nuclear translocation of a number of intracellular mediators [29]. Importantly, AX15836 boosted the effect of IVM in reducing A375 and HeLa cell proliferation (Fig. 6A,B), determining a reduction of cell viability by almost 75% in both cellular models. In the same experimental conditions, the evaluation of cell viability by cell counting showed a synergistic effect of IVM (10 μm) and AX15836 (1 μm) in reducing A375 and HeLa cell proliferation (Fig. S4B,C). Likewise, a synergistic effect of IVM and other ERK5 inhibitors (i.e. XMD8‐92 and JWG‐071) was obtained (Fig. S4D,E). Colony formation assay experiments performed with A375 cells showed that the combination of 10 μm IVM and 1 μm AX15836 halved the number of colonies formed by A375 cells, providing a synergistic effect (Fig. 6C), while each drug alone determined negligible (IVM) or no effect on colony formation. The same results were obtained in HeLa (Fig. 6D and Fig. S4F) and in SK‐Mel‐5 melanoma cells (Fig. S4G,H). In order to demonstrate that the increased effect of the combination between IVM and AX15836 in reducing cancer cell proliferation relies, at least in part, on ERK5, we used ERK5‐KO HUH‐7 cells (Fig. 6E). MTT assay showed that combined treatment with 5 μm IVM and 1 μm AX15836 markedly (by 35%) reduced cell viability on HUH‐7 parental cells, whereas it was ineffective in ERK5‐KO HUH‐7 cells (Fig. 6F).

**Fig. 7:**
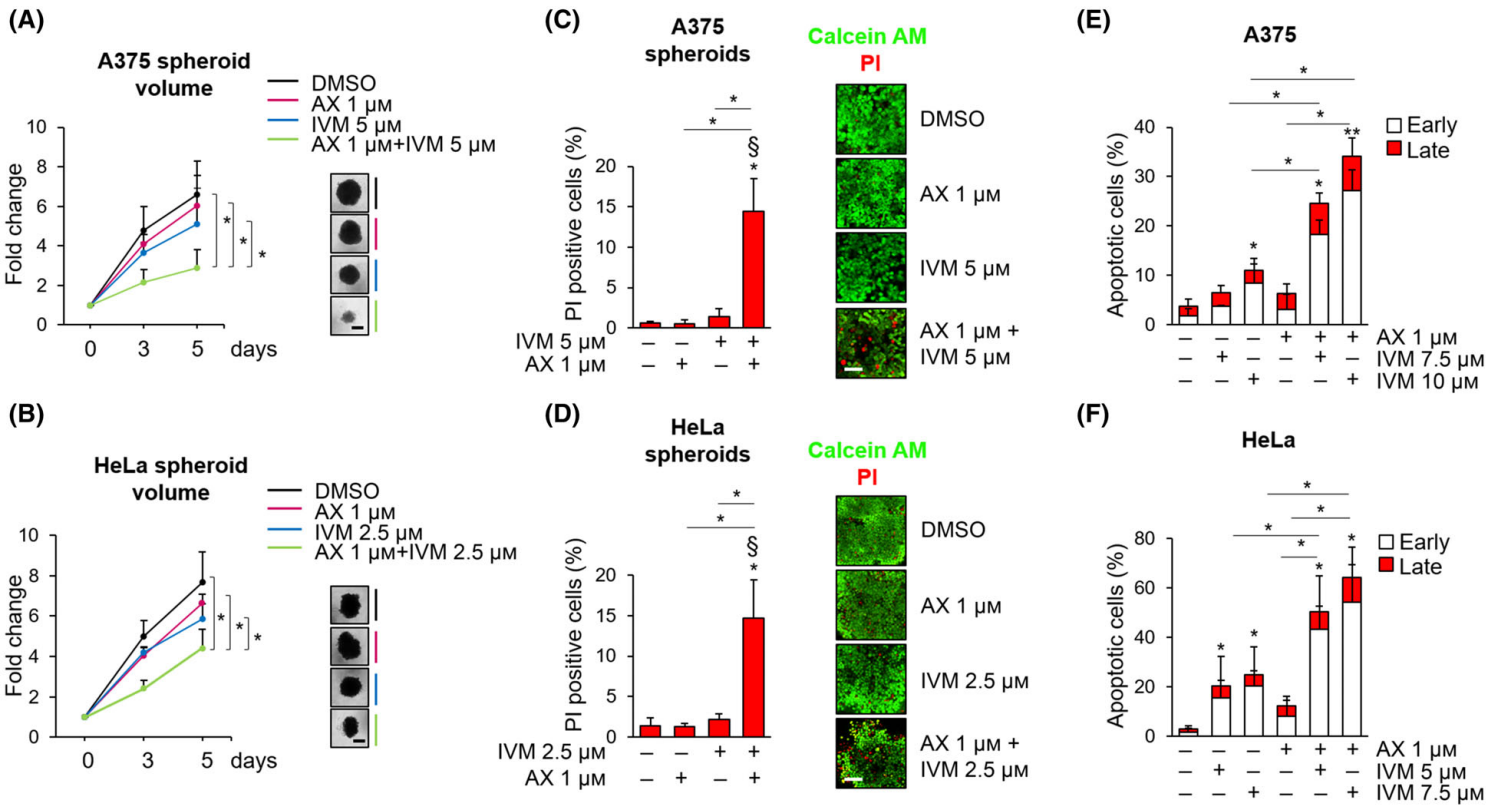
AX15836 induces cell death only in combination with ivermectin. (A) A375 spheroids were treated with Vehicle, 1 μm AX15836 (AX), 5 μm ivermectin (IVM) or with the combination of the two drugs. (B) HeLa spheroids were treated with Vehicle, 1 μm AX15836 (AX), 2.5 μm ivermectin (IVM) or with the combination of the two drugs. Graphs (A, B) show the quantification of spheroid volumes ± SD at different time points (0, 3 and 5 days) normalized for the time point 0 (*n* = 3). Representative images of spheroids taken at day 5 are shown. *P* values calculated using Student's *t*‐test refer to differences between the indicated conditions. *, *P* < 0.05. Scale bar: 400 μm. (C, D) Average values ± SD of the percentage of propidium iodide (PI) positive cells in A375 (C) and HeLa (D) spheroids stained with Calcein AM and propidium iodide (PI) after treatment with vehicle, ivermectin (IVM, 5 μm for A375, 2.5 μm for HeLa), AX15836 (AX) 1 μm or with the combination of the two drugs for 96 h (*n* = 3). Representative images of the experiments used for quantification, obtained with confocal analysis. *P* values calculated using Student's *t*‐test refer to differences with respect to vehicle‐treated or between the indicated conditions. *, *P* < 0.05. § indicates synergistic effect (bliss test > 0) with respect to single treatments. Scale bar: 80 μm. (E, F) A375 (E) and HeLa cells (F) were treated with vehicle (DMSO) or with 1 μm AX15836 (AX), or with 7.5 or 10 μm (E) or with 5 or 7.5 μm (F) ivermectin (IVM) or with the combination of them for 24 h. The graphs report average percentages ± SD of apoptotic cells evaluated through flow cytometry following Annexin V–propidium iodide staining (*n* = 3). The discrimination between early and late apoptosis has been determined on the basis of Annexin V‐positive/propidium iodide‐negative cells (early apoptosis) or Annexin V‐positive/propidium iodide‐positive (late apoptosis) cells. *P* values calculated using Student's *t*‐test refer to differences with respect to vehicle‐treated cells or between the indicated conditions. *, *P* < 0.05 and **, *P* < 0.01.

We then used A375 and HeLa spheroids to move to a 3D model of *in vitro* tumour growth. The combination of 1 μm AX15836 and IVM, at concentrations determining negligible effects when used alone, halved A375 and HeLa spheroid volumes (Fig. 7A,B). Quantification of dead cells in A375 and HeLa spheroids showed that the combination of AX15836 with IVM determined a robust increase of the percentage of PI‐positive cells with respect to vehicle‐treated spheroids with a synergistic effect (Fig. 7C,D).

With regard to the survival of A375 and HeLa cells grown in standard cultures (i.e. monolayers), we found that 1 μm AX15836 had negligible effects in inducing apoptosis in both cell lines (Fig. 7E,F). Treatment with IVM alone determined measurable apoptosis in A375 (10 μm) and HeLa (5 and 7.5 μm) cells. In combination with 1 μm AX15836, 7.5 or 10 μm IVM induced apoptosis in 25% and 35% (Fig. 7E), respectively, of A375 cells. In HeLa cells, the combination determined 50% to 60% apoptotic cells, depending on the dose of IVM used (5 or 7.5 μm IVM, respectively) (Fig. 7F). Of note, the major observed effects were attributable to early apoptosis.

**Fig. 8:**
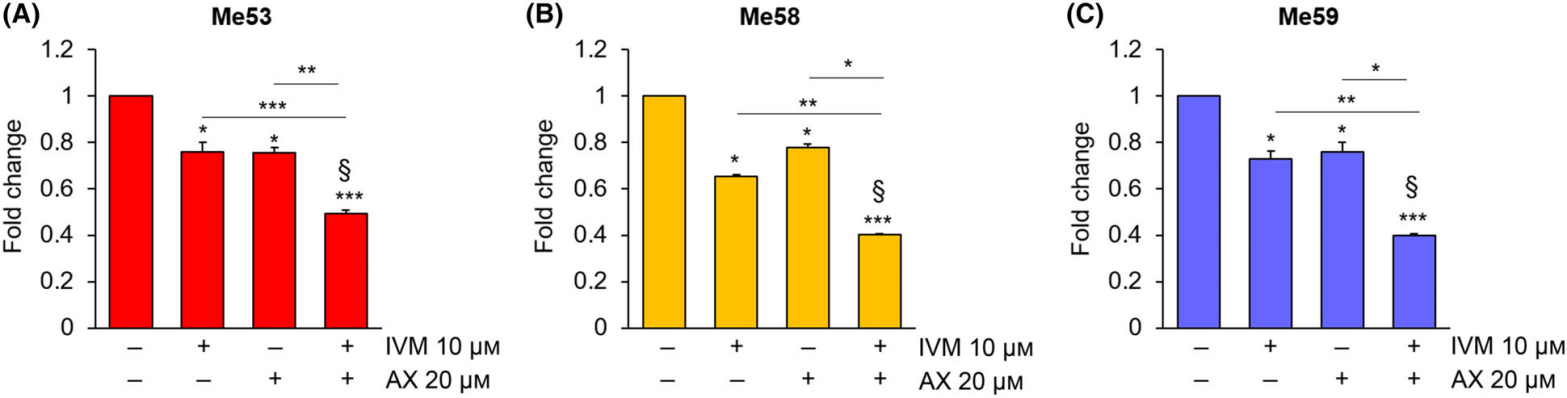
The combination of ivermectin with AX15836 reduces the viability of primary melanoma cells. (A–C) Me53 (A), Me58 (B) and Me59 (C) primary cells were treated with DMSO as a vehicle (−/−) or with 10 μm ivermectin (IVM), 20 μm AX15836 (AX) or with the combination of the two drugs for 72 h, and MTT assay was performed. The graphs report average data ± SD (*n* = 3). *P* values calculated using Student's *t*‐test refer to differences with respect to vehicle‐treated cells or between the indicated conditions. *, *P* < 0.05; **, *P* < 0.01; and ***, *P* < 0.001. § indicates synergistic effect (bliss test > 0) with respect to single treatments.

Finally, in short‐term cultures, the combination of IVM with AX15836 synergistically reduced the viability of primary melanoma cells by 50% (Me53) or 60% (Me58 and Me59), whereas treatment with each drug alone determined lower effects (Fig. 8A–C). The effect of the combination of AX15836 with a higher dose of IVM (13 μm) was even more pronounced, reaching 70% of reduction in all the primary cells used (Fig. S5). In this experimental setting, higher doses of AX15836 were used in keeping with the lower sensitivity to drugs of primary cultures with respect to that of cell lines in line with previous reports [30, 31]. However, we cannot exclude the occurrence of aspecific effects.

## 4 Discussion

ERK5 has a crucial role in the promotion of cancer cell growth [10], tied to its presence in the nucleus [7], where it can transcriptionally activate pro‐proliferative genes. Based on the presence of a cNLS and on the high molecular weight of this protein, it has been postulated that ERK5 shuttles into the nucleus through α/β importins [4, 16], although direct evidence has not been provided yet. The present study identifies importin β1 as the ERK5 nuclear transporter and proves IVM, an α/β1 importin inhibitor, as a drug able to prevent ERK5 nuclear translocation. The identification of the efficacy of IVM in preventing ERK5 translocation to the nucleus paves the way to a possible use of this drug to inhibit ERK5 functions linked to its presence in the nucleus, including the support of proliferative signals.

The involvement of importin β1 in the nuclear shuttling of ERK5 has been demonstrated using many approaches. First, western blot analysis provided evidence that both importin β1 KD and pharmacological inhibition of α/β1 importin using IVM prevented ERK5 nuclear translocation in routinely cultured cells and in unstimulated (i.e. in the absence of EGF or in the absence of MEK5DD) or stimulated (i.e. in the presence of EGF or MEK5DD) conditions. Second, immunofluorescence in confocal microscopy confirmed that IVM impairs EGF‐induced ERK5 nuclear shuttling. The above results were fully complemented by the super‐resolution microscopy approach. Indeed, the reduction of ERK5 molecules in the nucleus upon IVM treatment using this technical approach provides further evidence that α/β1 importin complexes are involved in ERK5 nuclear shuttling. The optimized technique will be also useful for future studies directed to characterize several aspects of ERK5 functions within the nucleus and of other MAPK. Finally, both co‐IP experiments and PLA provided evidence that ERK5 and importin β1 interact and that this interaction is further induced by EGF administration, while it is prevented by IVM treatment. However, whether the interaction of ERK5 with importin β1 is direct or mediated by an importin α subunit remains to be established [14].

It has been recently reported that AX15836, a small molecule ERK5 inhibitor that reduces ERK5 kinase activity, induces paradoxical activation of ERK5 by supporting its nuclear translocation and its transcriptional transactivation activity [26]. Accordingly, this study provides evidence that AX15836 is ineffective in reducing melanoma and cervical cancer cell proliferation. Additionally, similarly to what previously demonstrated in HeLa cells by others [27], we show here that AX15836 induces the increase of ERK5 nuclear translocation also in A375 melanoma cells. More importantly, despite IVM may have additional targets other than the α/β1 importin complexes [32, 33], it proved effective in preventing ERK5 nuclear shuttling upon AX15836 treatment. Moreover, in the presence of IVM, AX15836 reduced cancer cell viability and colony formation ability and increased apoptosis. Of note, IVM alone induced, at certain dosage, a reduction in cancer cell proliferation and an increase in the percentage of apoptotic cells. This is expected taking into consideration that importin β1 is involved in the nuclear translocation of pro‐proliferative factors other than ERK5 [34, 35, 36] and that IVM inhibits basal ERK5 nuclear translocation. Furthermore, AX15836 alone did not affect spheroid volume, whereas low doses of IVM slightly impacted on spheroid volume. However, they were able to reduce cancer cells spheroid volume when used in combination. The relatively low toxicity of IVM obtained in our experiments is in line with the fact that IVM is well tolerated by mice [37] and is an already FDA‐approved drug.

We also provide evidence that the synergistic effect between AX15836 and IVM is ERK5‐related. Indeed, in HUH‐7 ERK5 KO cells, the combination of IVM (used at a concentration ineffective as single drug) and AX15836 did not show any effect, while reducing cell viability in HUH‐7 parental cells. These results suggest that the effects observed using IVM and AX15836 in reducing cancer cell proliferation are not due to the overall IVM ability of inhibiting the nuclear translocation of pro‐proliferative factors, but to its ability to prevent ERK5 nuclear shuttling.

## 5 Conclusions

The identification of importin β1 as the nuclear transporter of ERK5 may be exploited for additional ERK5‐inhibiting strategies for cancer therapy. Indeed, targeting ERK5 nuclear translocation could provide a valid approach *per se* or in combination with ERK5 kinase inhibitors to counteract cancer cell proliferation. Furthermore, inhibition of ERK5 nuclear transport could be used in combination with other established targeted therapies to fight against those tumours in which ERK5 plays a relevant role.

## Conflict of interest

The authors declare no conflict of interest.

## Author contributions

ER conceived the study, designed and supervised the experimental procedures, analysed results, wrote the draft and revised the manuscript. ZL performed experimental work, designed experiments, analysed results and wrote the draft and revised the manuscript. ML and IT performed experiments, analysed results and revised the manuscript. AM, AT and LM performed experiments and analysed results. LG supervised, designed and performed the single‐molecule microscopy experiments and revised the manuscript. AVK analysed the single molecule results. BS designed experiments, analysed results and revised the manuscript, while MC designed the single‐molecule microscopy experiments and revised the manuscript.

## Supporting information


**Fig. S1.** Importin subunit beta‐1 silencing and its effect on ERK5 nuclear translocation in unstimulated conditions in HEK293T overexpressing ERK5.
**Fig. S2.** Ivermectin reduces the amount of ERK5 in the chromatin‐bound fraction, and inhibits ERK5 nuclear translocation in HEK293T cells overexpressing ERK5.
**Fig. S3.** ERK5‐HaloTag is detectable with an anti‐ERK5 antibody and translocates into the nucleus.
**Fig. S4.** Effects of combined ivermectin and ERK5i on the viability and colony‐formation ability in cancer cells.
**Fig. S5.** The combination of ivermectin with AX15836 reduces the viability of primary melanoma cells.

**Table S1.** List of the antibodies used and their application.

## Data Availability

Data sharing not applicable to this article as no datasets were generated or analysed during the current study.
