# Supplementary material for: Importin subunit beta‐1 mediates ERK5 nuclear translocation, and its inhibition synergizes with ERK5 kinase inhibitors in reducing cancer cell proliferation
Source: Mol Oncol. 2024 Jul 4;19(1):99–113. doi: 10.1002/1878-0261.13674 (PMC11705758; doi:10.1002/1878-0261.13674)
Supplement: Supplementary file 1 — Fig. S1. Importin subunit beta‐1 silencing and its effect on ERK5 nuclear translocation in unstimulated conditions in HEK293T overexpressing ERK5. Fig. S2. Ivermectin reduces the amount of ERK5 in the chromatin‐bound fraction, and inhibits ERK5 nuclear translocation in HEK293T cells overexpressing ERK5. Fig. S3. ERK5‐HaloTag is detectable with an anti‐ERK5 antibody and translocates into the nucleus. Fig. S4. Effects of combined ivermectin and ERK5i on the viability and colony‐formation ability in cancer cells. Fig. S5. The combination of ivermectin with AX15836 reduces the viability of primary melanoma cells. [file MOL2-19-99-s002.zip › Lombardi et al Suppl Information.pdf]

Importin subunit beta-1 mediates ERK5 nuclear translocation, and its inhibition synergizes with ERK5 kinase inhibitors in reducing cancer cell proliferation

Zoe Lombardi[1], Lucia Gardini[2,3]*, Anatolii V. Kashchuk[3,4]*, Alessio Menconi[1], Matteo Lulli[1], Ignazia Tusa[1], Alessandro Tubita[1], Luisa Maresca[5], Barbara Stecca[5], Marco Capitanio[3,4], Elisabetta Rovida[1]

[1]Department of Clinical and Experimental Biomedical Sciences, University of Florence, Florence, Italy.
[2]National Institute of Optics, National Research Council, Florence, Italy
[3]European Laboratory of Non-Linear Spectroscopy (LENS), Sesto Fiorentino, Italy.
[4]Department of Physics and Astronomy, University of Florence, Sesto Fiorentino, Italy
[5]Core Research Laboratory - Institute for Cancer Research and Prevention (ISPRO), Florence, Italy.

*These two authors equally contributed to the work

Supplementary Figure legends

Supplementary Figure 1. *Importin subunit beta-1 silencing and its effect on ERK5 nuclear translocation in unstimulated conditions in HEK293T overexpressing ERK5.* A375 (**A**) and HEK293T (**B**) cells transfected with two siRNA targeting KPNB1 (siKPNB1-1 or siKPNB1-2) or with non-targeting siRNA (siNT) were lysed after 72 hours and Western Blot was performed with the indicated antibodies. Migration of molecular weight markers is indicated on the left (kDa). The graphs show average densitometric values of importin β1 protein levels normalized for tubulin content. P values refer to differences with respect to siNT control cells. *, P< 0.05; **, P < 0.01. (**C**) HEK293T overexpressing ERK5 were transfected with KPNB1-targeting (siKPNB1-2) or with non-targeting control (siNT) siRNA and lysed. Western Blot was performed on cytoplasmic or nuclear extracts with the indicated antibodies. Migration of molecularweight markers is indicated on the left (kDa). The graph shows average densitometric values of nuclear ERK5 protein levels normalized for fibrillarin content from three independent experiments. P values refer to differenceswith respect to siNT control cells. *, P < 0.05.

Supplementary Figure 2. *Ivermectin reduces the amount of ERK5 in the chromatin-bound fraction, and inhibits ERK5 nuclear translocation in HEK293T cells overexpressing ERK5.* **A**) HEK293T cells overexpressing ERK5 and MEK5DD and A375 cells were treated with the indicated doses of IVM for 24h. Cells were then lysed and Western Blot was performed using chromatin-bound extracts with the indicated antibodies. Migration of molecular weight markers is indicated on the left (kDa). IVM, ivermectin. **B**) HEK293T ERK5+ or ERK5+/MEK5DD+ cells were treated for 24h with 10 μM IVM and lysed. Western Blot was performed on cytoplasmic or nuclear extracts with the indicated antibodies. Migration of molecularweight markers is indicated on the left (kDa). The graph shows average densitometric values of nuclear ERK5 protein levels normalized for fibrillarin content from three independent experiments. P

values refer to differenceswith respect to untreated HEK293T cells (*) or between the indicated conditions (#). *,# $P < 0.05$. l.e., low exposure.

Supplementary Figure 3. ERK5-*HaloTag is detectable with an anti-ERK5 antibody and translocates into the nucleus.* **A**) HEK293T cells transfected with ERK5- or with ERK5-HaloTag-containing vectors were lysed and Western Blot was performed with the indicated antibodies. Migration of molecular weight markers is indicated on the left (kDa). ERK5-HaloTag is detectable with an anti-ERK5 antibody at a higher molecular weight compared to ERKWT. **B**) HeLa cells transfected with ERK5-HaloTag and MEK5DD were treated with DMSO (-/-) used as vehicle or 5 μM IVM for 24h and then lysed. Western Blot was then performed on whole cell lysates with the indicated antibodies. Migration of molecular weight markers is indicated on the left (kDa). **C**) Highlight of the edges used to draw the nuclear masks that are easily visible in bright-field images of HeLa cells.

Supplementary Figure 4. *Effects of combined ivermectin and ERK5i on the viability and colony-formation ability in cancer cells.* **A**) Sk-Mel-5 cells were treated with increasing concentrations of AX15836 and MTT assay was performed after 72h. **B, C)** A375 (**B**) or HeLa (**C**) cells were treated with DMSO used as vehicle (-/-) or 10 μM IVM, or 1 μM AX15836 (AX) or with the combination of the two drugs for 72h and the number of viable cells determined using trypan blue exclusion test. P values refer to differences with respect to vehicle-treated cells (*) or between the indicated conditions (#). *,# $P < 0.05$; **,## $P < 0.01$. § indicate synergistic effect (bliss test >0). **D-E)** A375 cells were treated with DMSO used as vehicle (-/-), or 10 μM IVM or 5 μM JWG-071 (**D**) or XMD8-92 (**E**) or with their combination for 72 hours and the number of viable cells determined using trypan blue exclusion test. Histograms represent mean ± SD from three independent experiments. P values refer to differences with respect to vehicle-treated cells (*) or between the indicated conditions (#). *,# $P < 0.05$; **,## P

< 0.01. § indicate synergistic effect (bliss test >0). **F**) Colony formation assay was performed with HeLa cells treated with DMSO used as vehicle (-/-), IVM or AX15836 (AX) or with their combination at the indicated concentrations for 10 days. P values refer to differences with respect to vehicle-treated cells (*) or between the indicated conditions (#). *,$^{\#}$ P < 0.05; **, P < 0.01. § indicates synergistic effect (bliss test >0). **G**) Sk-Mel-5 cells were treated with increasing concentrations of AX15836 or IVM or their combination and MTT assay was performed after 72h. **H**) Colony formation assay was performed with Sk-Mel-5 cells treated with DMSO used as vehicle (-/-), IVM or AX15836 (AX) or with their combination at the indicated concentrations for 10 days. P values refer to differences with respect to vehicle-treated cells (*) or between the indicated conditions (#). *,$^{\#}$ P < 0.05; $^{\#\#}$, P < 0.01. § indicates synergistic effect (bliss test >0).

Supplementary Figure 5. *The combination of ivermectin with AX15836 reduces the viability of primary melanoma cells*. **A-C)** Me59, Me53 and Me58 primary melanoma cells were treated with DMSO used as vehicle (-/-), IVM, AX15836 (AX) or with the combination of the two drugs for 72h and MTT assay was performed. P values refer to differences with respect to vehicle treated cells (*) or between indicated conditions (#). *,$^{\#}$ P < 0.05; **,$^{\#\#}$ P < 0.01; *** P < 0.001. § indicate synergistic effect (bliss test >0).
