# Supplementary material for: Importin subunit beta‐1 mediates ERK5 nuclear translocation, and its inhibition synergizes with ERK5 kinase inhibitors in reducing cancer cell proliferation
Source: Mol Oncol. 2024 Jul 4;19(1):99–113. doi: 10.1002/1878-0261.13674 (PMC11705758; doi:10.1002/1878-0261.13674)

Supplementary Figure 1

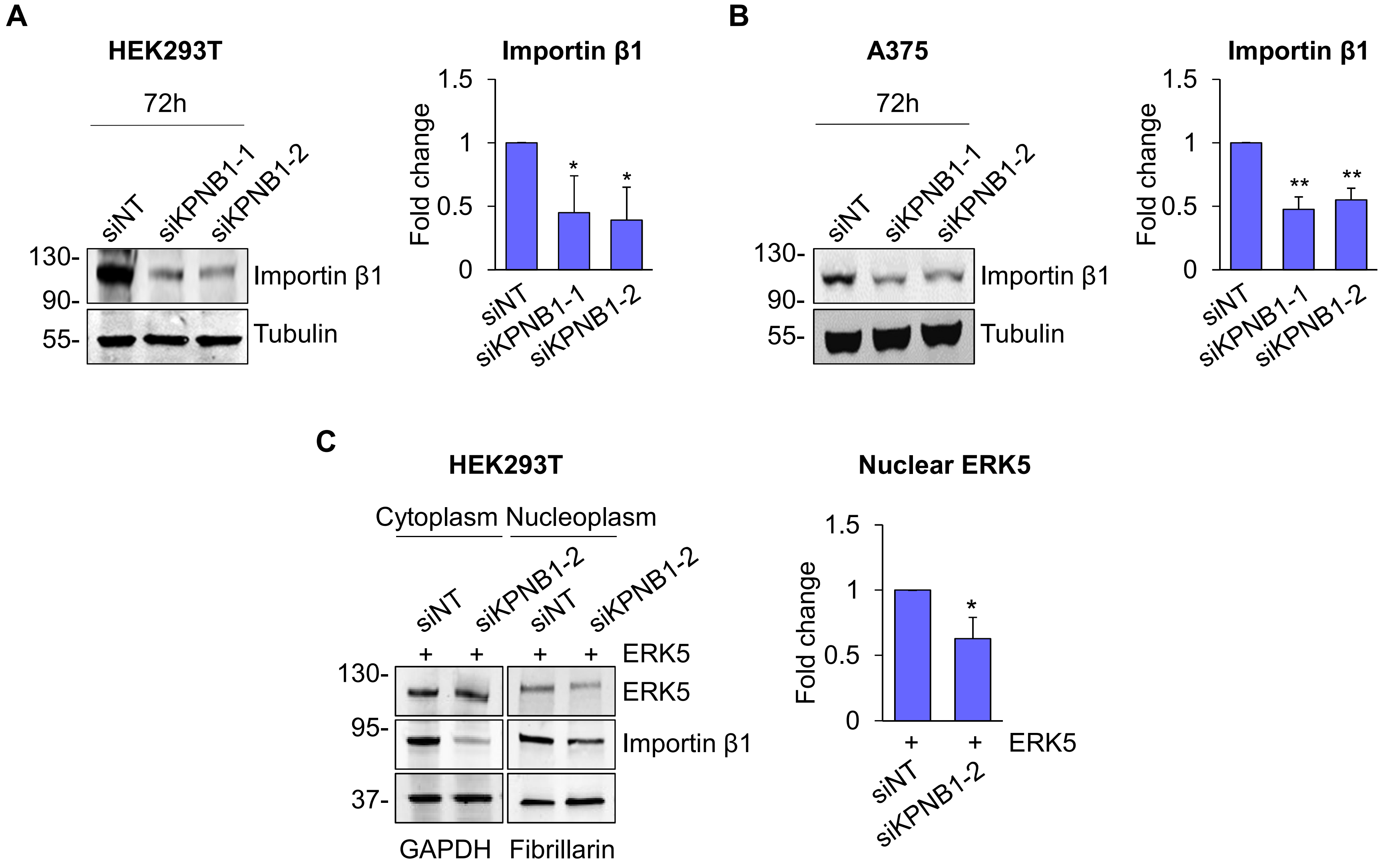

Supplementary Figure 2

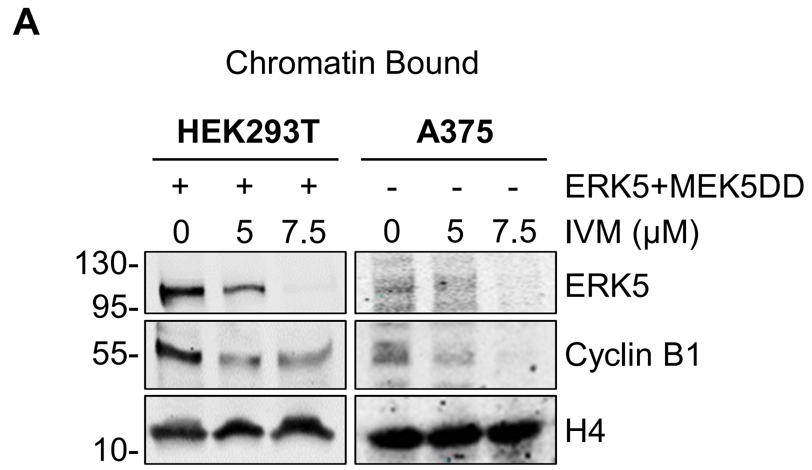

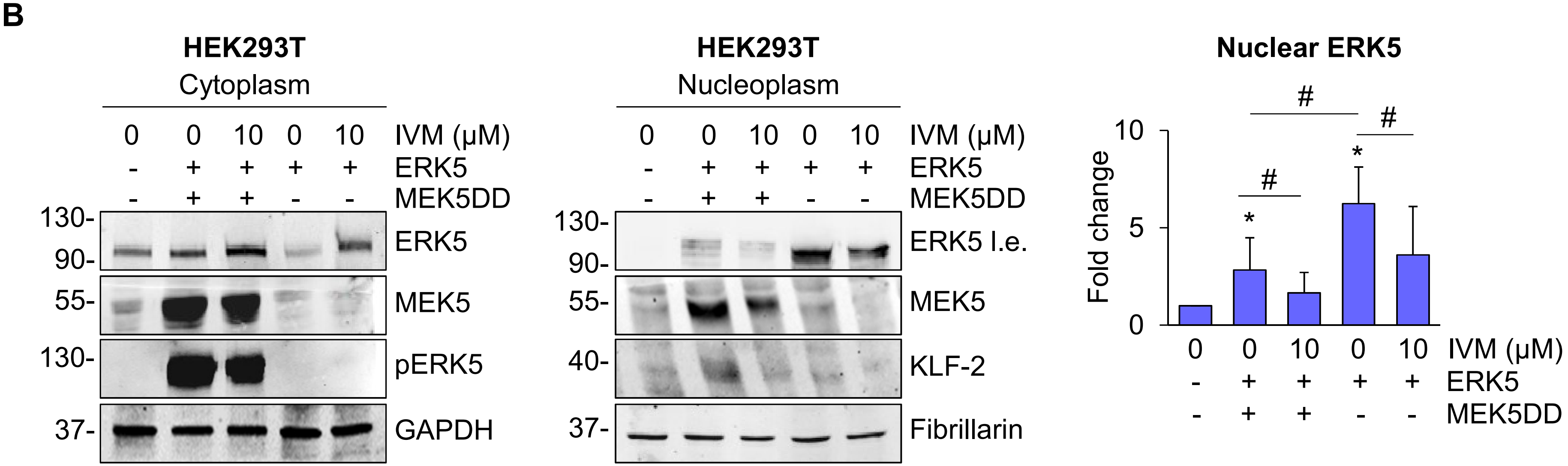

Supplementary Figure 3

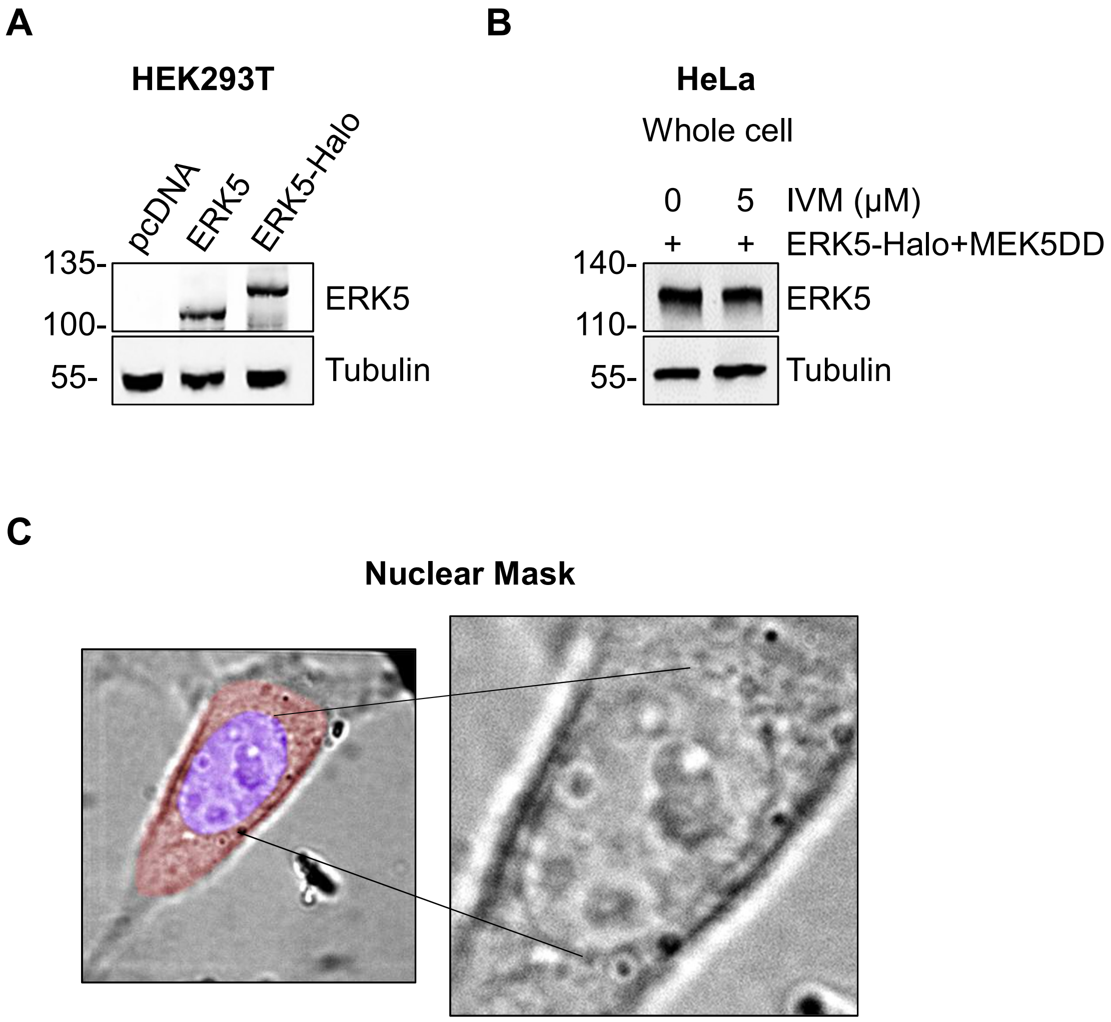

Supplementary Figure 4

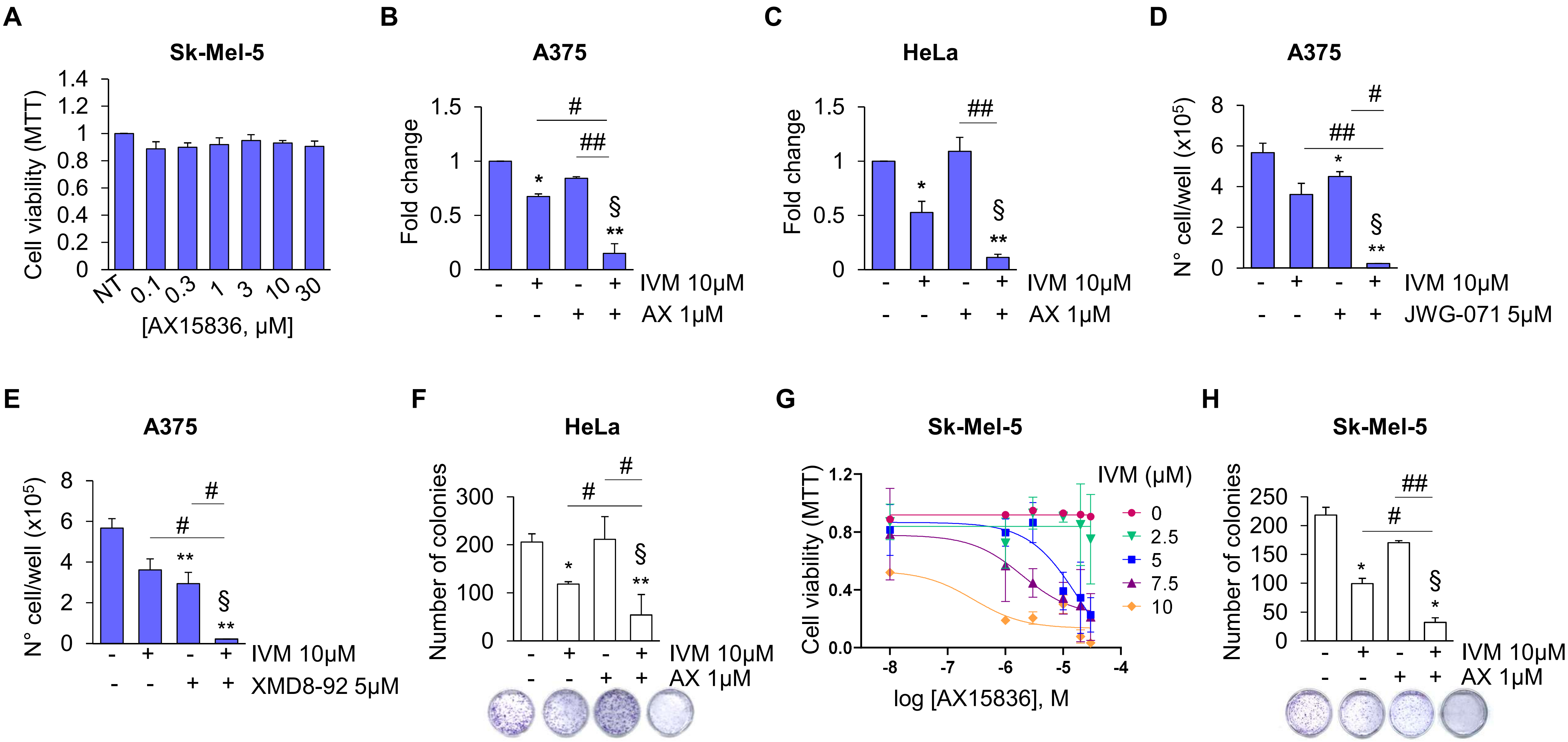

Supplementary Figure 5

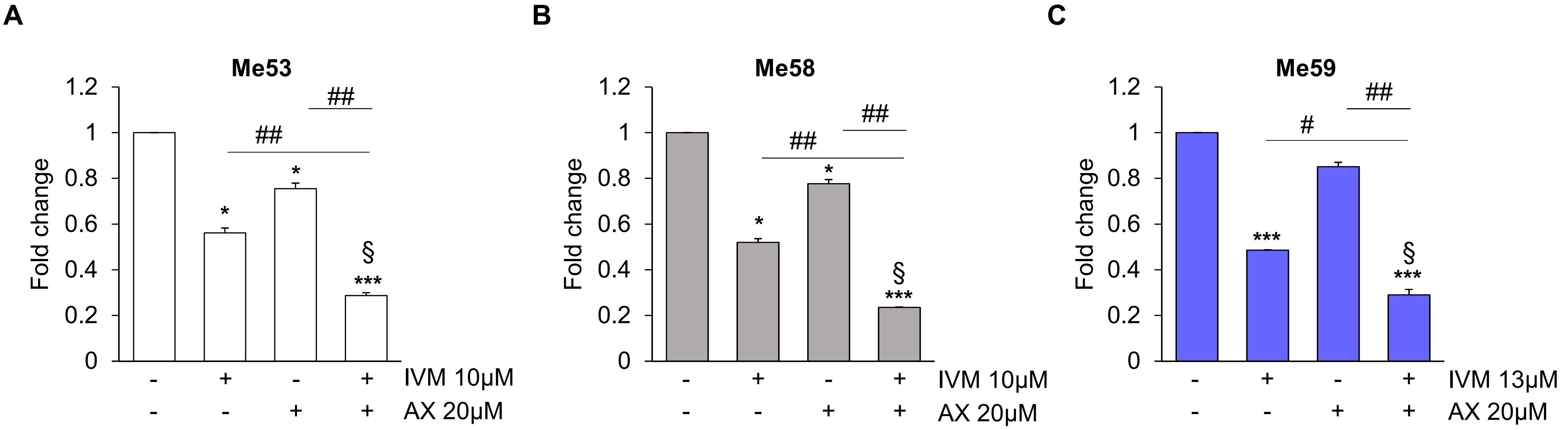

Supplement: Supplementary file 1 — Fig. S1. Importin subunit beta‐1 silencing and its effect on ERK5 nuclear translocation in unstimulated conditions in HEK293T overexpressing ERK5. Fig. S2. Ivermectin reduces the amount of ERK5 in the chromatin‐bound fraction, and inhibits ERK5 nuclear translocation in HEK293T cells overexpressing ERK5. Fig. S3. ERK5‐HaloTag is detectable with an anti‐ERK5 antibody and translocates into the nucleus. Fig. S4. Effects of combined ivermectin and ERK5i on the viability and colony‐formation ability in cancer cells. Fig. S5. The combination of ivermectin with AX15836 reduces the viability of primary melanoma cells. [file MOL2-19-99-s002.zip › Supplementary Figures.pdf]
