# Supplementary material for: Importin subunit beta‐1 mediates ERK5 nuclear translocation, and its inhibition synergizes with ERK5 kinase inhibitors in reducing cancer cell proliferation
Source: Mol Oncol. 2024 Jul 4;19(1):99–113. doi: 10.1002/1878-0261.13674 (PMC11705758; doi:10.1002/1878-0261.13674)
Supplement: Supplementary file 2 — Table S1. List of the antibodies used and their application. [file MOL2-19-99-s001.pdf]

**Supplementary Table S1.** *List of the antibodies used and their application.*

| | | | | |
|---|---|---|---|---|
| Importin β1 | WB | Rabbit monoclonal | #8673 | Cell Signaling Technology, Danvers, MA, USA |
| Fibrillarin | WB | Mouse monoclonal | sc-374022 | Santa Cruz Biotechnology, Santa Cruz, CA, USA |
| ERK5 | WB | Rabbit polyclonal | #3372 | Cell Signaling Technology, Danvers, MA, USA |
| ERK5 | IF | Mouse monoclonal | sc-398015 | Santa Cruz Biotechnology, Santa Cruz, CA, USA |
| pERK5-T218/Y220 | WB | Rabbit polyclonal | #3371 | Cell Signaling Technology, Danvers, MA, USA |
| KLF-2 | WB | Rabbit monoclonal | #15306 | Cell Signaling Technology, Danvers, MA, USA |
| MEK5 | WB | Mouse monoclonal | sc-365198 | Santa Cruz Biotechnology, Santa Cruz, CA, USA |
| Importin β1 | IF | Rabbit polyclonal | PA5-83110 | ThermoFisher Scientific, Waltham, MA, USA |
| Cyclin B1 | WB | Rabbit monoclonal | sc-245 | Santa Cruz Biotechnology, Santa Cruz, CA, USA |
| H4 | WB | Rabbit monoclonal | #13919 | Cell Signaling Technology, Danvers, MA, USA |
| α Tubulin | WB | Mouse monoclonal | sc-32293 | Santa Cruz Biotechnology, Santa Cruz, CA, USA |
| GAPDH | WB | Mouse monoclonal | sc-47724 | Santa Cruz Biotechnology, Santa Cruz, CA, USA |
| IRDye 800CW | WB | Goat anti-rabbit | 926-32211 | LI-COR Biosciences, Lincoln, NE, USA |
| IRDye 800CW | WB | Goat anti-mouse | 926-32210 | LI-COR Biosciences, Lincoln, NE, USA |
| IRDye 680RD | WB | Goat anti-rabbit | 926-68071 | LI-COR Biosciences, Lincoln, NE, USA |
| IRDye 680RD | WB | Goat anti-mouse | 926-68070 | LI-COR Biosciences, Lincoln, NE, USA |
